# Artificial Intelligence to Get Insights of Multi-Drug Resistance Risk Factors during the First 48 Hours from ICU Admission

**DOI:** 10.3390/antibiotics10030239

**Published:** 2021-02-27

**Authors:** Inmaculada Mora-Jiménez, Jorge Tarancón-Rey, Joaquín Álvarez-Rodríguez, Cristina Soguero-Ruiz

**Affiliations:** 1 Department of Signal Theory and Communications, Telematics and Computing Systems, Rey Juan Carlos University, 28943 Fuenlabrada, Madrid, Spain; inmaculada.mora@urjc.es (I.M.-J.); j.tarancon.2016@alumnos.urjc.es (J.T.-R.); 2 University Hospital of Fuenlabrada, 28943 Fuenlabrada, Madrid, Spain; joaquin.alvarez@salud.madrid.org

**Keywords:** artificial intelligence, machine learning, feature selection, risk factors, multi-drug resistance, Intensive Care Unit, antibiotics

## Abstract

Multi-drug resistance (MDR) is one of the most current and greatest threats to the global health system nowadays. This situation is especially relevant in Intensive Care Units (ICUs), where the critical health status of these patients makes them more vulnerable. Since MDR confirmation by the microbiology laboratory usually takes 48 h, we propose several artificial intelligence approaches to get insights of MDR risk factors during the first 48 h from the ICU admission. We considered clinical and demographic features, mechanical ventilation and the antibiotics taken by the patients during this time interval. Three feature selection strategies were applied to identify statistically significant differences between MDR and non-MDR patient episodes, ending up in 24 selected features. Among them, SAPS III and Apache II scores, the age and the department of origin were identified. Considering these features, we analyzed the potential of machine learning methods for predicting whether a patient will develop a MDR germ during the first 48 h from the ICU admission. Though the results presented here are just a first incursion into this problem, artificial intelligence approaches have a great impact in this scenario, especially when enriching the set of features from the electronic health records.

## 1. Introduction

Antimicrobials were one of the most outstanding inventions in the health sector since penicillin was discovered in 1928, aimed at controlling bacterial infections [1]. An antimicrobial can be defined as a substance that kills or inhibits the growth of microorganisms (bacteria, fungi or parasites) [2]. Depending on the microorganism they attack, antimicrobials are classified as: antibiotics, antivirals, fungal and antiprotozoal agents [3]. They constitute one of the most important tools both for prevention and treatment of infectious diseases in humans and animals, and therefore are extremely important in conventional medicine. Sometimes antimicrobials do not perform the desired action on the microorganism. When a microorganism changes from being susceptible to an antimicrobial to being unaffected by it, it is called antimicrobial resistance [4]. Antimicrobial resistance, defined as the ability of bacteria to survive against chemical agents designed to kill them [5], is not necessarily limited to a single family of antibiotics. In fact, multi-drug resistance (MDR) occurs when a bacterium becomes resistant to many antibiotics [6]. The growing problem of antimicrobial resistance poses a global threat in getting worse the effectiveness of these drugs [7]. The cost associated with MDR is becoming alarming at present, making it necessary to push actions to tackle this problem [8]. The antibiotic resistance is not a major problem for bacteria with innate resistance. However, the induced or developed resistances may lead to complications in the treatment of infectious diseases which are curable by antibiotic therapy.

There are scenarios in which the development of infections caused by antimicrobial resistance is more likely to occur. Health centers stand out among these scenarios, due to the continuous presence of germs and use of antibiotics. In addition, half of infections arising in hospitals are originated in the Intensive Care Unit (ICU) [4,9], probably due to the serious medical state of the patients in this unit. To determine whether the patient has been infected by an MDR bacteria in the ICU, it is usual to consider the result of a culture performed in a time interval of 48 h from the patient ICU admission, which corresponds to the time required to get the result of the culture. If the result of this culture is positive, it is conventionally considered that the antimicrobial resistance has not been acquired in the ICU.

Machine learning (ML), which has been applied in several clinical scenarios [10,11] by extracting knowledge from electronic health records (historical data), emerges in this study as a computational tool to create models anticipating the result of the culture. ML encompasses statistical and computational techniques to create models by learning the underlying relationships among a set D of *N* samples (historical data) [12,13]. For the learning task considered in this paper, each sample in D is composed of: input variables, arranged in a vector of *d* features x; and the corresponding target output (class), denoted by a one-dimensional variable *y* (encoded by ‘0’ to identify non-MDR episodes and ‘1’ for MDR ones), i.e., $D={\left\{\left(x^{\left(n\right)},y^{\left(n\right)}\right)\right\}}_{n=1}^{N}$. Since non-MDR episodes are usually more frequent than MDR ones, which is good from a clinical viewpoint, it causes some difficulties for training data-driven models with good generalization capabilities [14].

**Figure 1:**
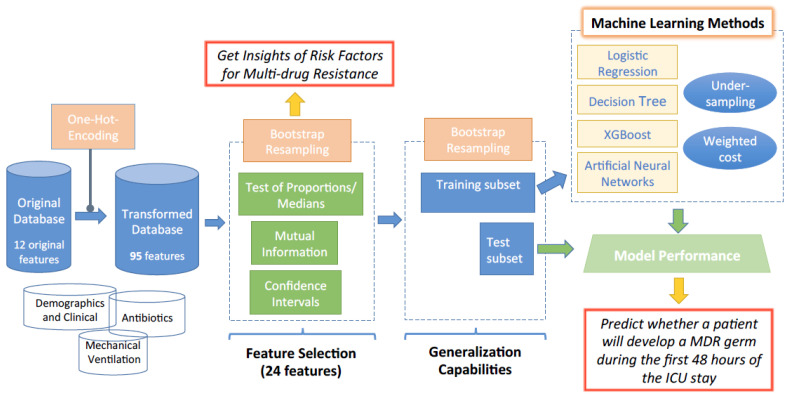
Workflow diagram of the proposed methodology to get insights of multi-drug resistance (MDR) risk factors and to predict whether a patient will develop an MDR germ during the first hours from the Intensive Care Unit (ICU) admission.

ML techniques have been used in different studies to determine whether a bacterium will be resistant to different families of antimicrobials during the stay of the patients in the ICU [15,16,17,18,19]. In this paper, we focus on getting insights about the risk factors and prediction of MDR during the first 48 h from the ICU admission. Towards that end, we follow the workflow depicted in Figure 1. Since the database for knowledge extraction is composed by categorical features, a pre-processing stage is required for ML algorithms to deal with these types of data. In this work, a One-Hot-Encoding strategy is carried out for categorical features. Thus, a binary feature is created for each category, ending up with 95 features. The increase in the dimension of the feature space advocates the application of Feature Selection (FS) strategies. Specifically, three FS approaches are considered to identify an appropriate final subset of features (24 features). This subset is obtained as the union of the features identified by each FS strategy. The selected features are considered to train several models for MDR prediction. Two strategies for dealing with imbalanced classes (undersampling and weighted cost) are evaluated. The results obtained in this work will help clinicians to identify whether a patient will develop an MDR germ during the first 48 h from the ICU admission.

The rest of the paper is organized as follows: Section 2 and Section 3 present the statistical approaches used in the FS and learning process, respectively. The database is described in Section 4, while the predictive results are shown in Section 5. Finally, main conclusions and discussion are drawn in Section 6.

## 2. Feature Selection Using Bootstrap

In order to carry out an adequate data analysis, it is convenient to develop an FS strategy to identify informative features in relation to the target output. An additional advantage of FS is the potential reduction in the model complexity, making easier the model interpretability too [20].

To get a statistical characterization of the goodness of the selected features, we have considered the use of bootstrap. Bootstrap is a technique allowing us to obtain non-parametric statistics on a population from multiple resamples [21,22,23]. This approach can be used to estimate confidence intervals, to perform hypothesis tests or to evaluate the performance of a specific ML scheme [24,25]. As it will be presented later, bootstrap will be considered here both in the feature selection and in the learning process (see Figure 1 for details).

There are different FS approaches, categorized as filter, wrapper and embedded methods [26]. On the one hand, filter methods select features without considering a predictive model, i.e., do not consider any inductive algorithm. On the other hand, wrapper and embedded approaches somehow take into account the model created by following a ML strategy. In particular, the wrapper methods evaluate the use of different subsets of features with a specific ML model, selecting the subset providing the best performance [27]. Finally, the embedded methods closely nest the FS process and the model design. Thus, the subset of features provided by the embedded methods are found while training the ML model [28]. In this paper, we focus on different filter approaches: (1) test of proportions [29] for binary features and test of medians [30] for numerical features; (2) mutual information [31]; and (3) a test based on confidence intervals [32]. As previously indicated, these FS techniques have been used in combination with bootstrapping.

### 2.1. Test of Proportions

It aims to test the validity of a null hypothesis ($H_{0}$) against an alternative one ($H_{1}$) using a set of *N* samples from a population with MDR and non-MDR episodes. For each feature, $p_{0}$ represents the proportion of non-MDR episodes with an active value (‘1’) in this feature, whereas $p_{1}$ corresponds to this proportion for MDR episodes. Thus, the hypothesis test can be stated as:

(1) $$H_{0}:p_{1}=p_{0}\;vs.\;H_{1}:p_{1}\neq p_{0},$$

where the $H_{1}$ hypothesis is accepted when proportions of active feature is statistically different in both populations [29]. For this purpose, the test statistic (*z*) and the associated *p*-value are calculated [33].

Since bootstrapping is used, the average of the *p*-values obtained in the resamples is computed and compared with the significance level for rejecting $H_{0}$ by taking into account a level of significance α.

### 2.2. Test of Medians

For numerical features, the Mood’s median test [30] was considered to check whether the medians of both populations (MDR and non-MDR episodes during the first 48 h in ICU) are the same. According to the bootstrapping technique used, the median value is calculated taking into account all values in the resample, regardless of the class they belong to. Then, samples are divided into two groups, depending on the corresponding value (for that feature) is above or below the median [34,35]. This allows us to obtain a contingency table of frequencies, made up of four cells because it is also considered the class each sample is associated to (MDR and non-MDR). Finally, the contingency table is used to calculate the Pearson’s Chi-squared test statistic ($\chi^{2}$) for each resample [36]. The mean of the $\chi^{2}$ values is compared with the significance level α to determine the validity of the null hypothesis.

### 2.3. Mutual Information

The mutual information (MI) value [37,38], which is based on the Shannon entropy [39], has also been obtained for FS purposes. The Shannon entropy H(.) of a discrete random variable (r.v.) *X* taking values x∈X is defined as H(X) = $-\sum_{x\in X}p\left(x\right)\log\left(p\left(x\right)\right)$, where p(x) is Pr{X=x}, and corresponds to the probability of the r.v. *X* to have the value *x*. For a continuous r.v. *X*, the definition of H(.) is similar, but p(x) corresponds now to the density function of the r.v. and the summation symbol is replaced by the integral one. The entropy H(X) measures the uncertainty of the r.v. *X* [31], and a high value of H(X) is associated with a uniform distribution of *X*. The MI between two random variables *X* and *Y* measures the shared information between them, and is computed as MI(X,Y)=H(X)−H(X|Y)=H(Y)−H(Y|X)=MI(Y,X). In other words, MI is the amount of information that the r.v. *X* has about the r.v. *Y*. In the context of FS, MI(X,Y) allows to determine the degree of dependency (or the absence of dependency) between the feature represented by the r.v. *X* and the target output represented by the r.v. *Y* [31]. Thus, a value of zero for the MI indicates absence of dependency among both variables, while higher values of MI are associated with greater dependence between them [37,38]. Since a bootstrapping technique is considered, for each resample and feature, the MI is computed. Then, the average of the MI values is used for feature selection.

### 2.4. Confidence Interval

**Figure 2:**
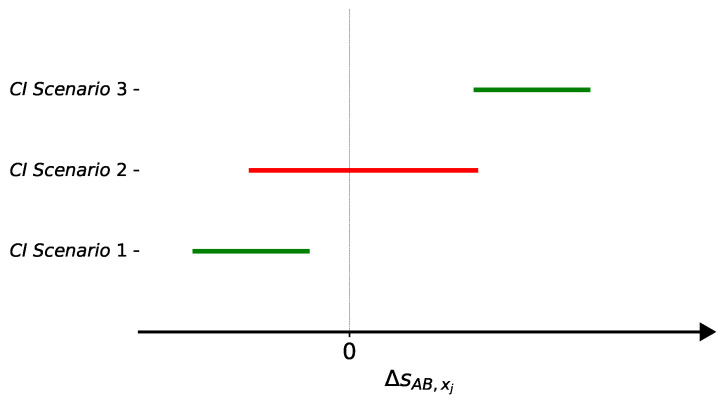
Three possible scenarios for the confidence interval (CI) of $\Delta s_{AB,x_{j}}$. The feature $x_{j}$ will be selected in Scenarios 1 and 3.

In order to explore a different indicator about the relevance of each feature, we compute the confidence interval (CI) of the difference between certain statistics when resampling (with no repetition) episodes of both classes (MDR and non-MDR) [21,29]. In particular, *R* resamplings of the original set D are considered, providing populations of the two classes: Population A for MDR and Population B for non-MDR episodes. In the case of each numerical feature $x_{j}$ and resample *r*, the medians $m_{A,x_{j}}\left(r\right)$ and $m_{B,x_{j}}\left(r\right)$ are computed for each population. Then, the difference between both statistics $\Delta m_{AB,x_{j}}\left(r\right)=m_{A,x_{j}}\left(r\right)-m_{B,x_{j}}\left(r\right)$ is calculated. When $x_{j}$ is a binary feature, the difference between the proportion of samples with value ‘1’ in each population is computed as $\Delta p_{AB,x_{j}}\left(r\right)=p_{A,x_{j}}\left(r\right)-p_{B,x_{j}}\left(r\right)$, with r=1,⋯R. Whatever the feature $x_{j}$, the *R* values of these differences are considered to estimate the corresponding 95% confidence interval, i.e., $CI_{\Delta s_{AB},x_{j}}$, where *s* denotes the considered statistic for a particular feature $x_{j}$. Then, a statistical hypothesis test is performed to select those features such that $CI_{\Delta s_{AB},x_{j}}$ does not overlap the zero value. Figure 2 illustrates three different scenarios for the CI of $\Delta s_{AB,x_{j}}$. In both Scenario 1 and Scenario 3, since the CI does not overlap the zero value, the feature $x_{j}$ is selected. However, in Scenario 2 the feature $x_{j}$ is not selected according to this criterion.

## 3. Machine Learning Methods

The use of ML techniques allows us to tackle a classification task as the one addressed in this work by creating a data-driven model. There have been proposed in the ML literature many techniques to create these models [40], by optimizing a cost function L. This function quantifies the “difference” between the target output and the output provided by the model. We explore in this paper several techniques with different complexity, interpretability and generalization ability: Logistic Regression, Decision Trees, XGBoost and Artificial Neural Network.

### 3.1. Evaluation of the Generalization Capability

Data-driven models are evaluated considering samples not used during their design [22]. Thus, the set of *N* available samples in D is randomly partitioned in two independent subsets: training and test subset. In this work, the usual 80/20% (training/test) proportion has been applied. The training subset is used for designing the model, while the generalization model capability (model performance) is estimated with the test subset. Several figures of merit have been considered to evaluate the model performance: accuracy, sensitivity, specificity and the area under the Receiver Operating Characteristic (ROC), also named AUC-Score [41].

The learning process associated with each ML technique is controlled by different hyperparameters. In this paper, hyperparameters have been tuned following a *K*-fold cross validation (CV) strategy [42]. In *K*-fold CV, the training subset is randomly divided into *K* folds or disjoints subsets, all of them of the same size (approximately). Then, each subset is successively used to evaluate the model trained with the remaining (K−1) subsets [40]. In this paper, the best hyperparameters configuration is selected as the one providing the highest performance following a 5-fold CV approach. The AUC-Score has been the figure of merit chosen for tuning the hyperparameters, since it provides a trade-off between sensitivity and specificity.

To avoid the potential bias when considering just one random training/test subset, it is convenient to repeat the partitioning several times, evaluating the performance of each classifier with the corresponding test subset. In this work, 50 random partitions of the training/test subset have been performed.

### 3.2. Learning with Imbalanced Classes

Since the number of non-MDR episodes in the first 48 h is much higher than the number of MDR episodes in the same period, learning approaches can provide models biased to get better performance for the majority class, therefore leading to a poor generalization. To overcome this difficulty in the learning process (class imbalance problem), several strategies can be implemented.

Learning with imbalanced classes will be carried out both by undersampling the majority class and by modifying the cost function to weight differently the misclassification errors in training [14]. *Undersampling* is intended to reduce the number of training samples of the majority class by randomly selecting a subset of samples, thus satisfying the objective of class balancing [43]. With this approach, it is possible to discard samples which could help to create a model with better generalization properties. Therefore, to avoid that the model performance is biased by one particular partition, the undersampling process and subsequent model construction are performed several times (50 in this paper).

Another approach to deal with imbalanced classes is to incorporate a priori information in the cost function. The idea behind this approach is to use all the available samples, but considering in the cost function a different weight $\beta_{0}$ and $\beta_{1}$ for the non-MDR and MDR episodes, respectively. That is, misclassifications are not equally weighted in the cost function L:

(2) $$L=-\frac{1}{N_{t}}\sum_{i=1}^{N_{t}}\left(\beta_{0}y^{\left(i\right)}\log\left({\hat{y}}^{\left(i\right)}\right)+\beta_{1}\left(1-y^{\left(i\right)}\right)\log\left(1-{\hat{y}}^{\left(i\right)}\right)\right),$$

where $N_{t}$ is the number of training samples, with $N_{t}=N_{0}+N_{1}$ and $N_{0}$/$N_{1}$ the number of non-MDR/MDR episodes, $\beta_{0}=\frac{N_{t}}{2*N_{0}}$ and $\beta_{1}=\frac{N_{t}}{2*N_{1}}$.

### 3.3. Logistic Regression

Logistic Regression (LR) is a parametric approach estimating the target value as a linear combination of the input features [44,45]. Therefore, the separation between classes corresponds to a hyperplane in *d* dimensions. To allow better generalization capabilities, coefficients $w\;=\;[w_{1},w_{2},w_{3},\cdots ,w_{d}]$ and *b* (bias) of the linear model are found by optimizing the following regularized cost function:

(3) $$L=\frac{1}{2}ww^{T}+C\sum_{i=1}^{N_{t}}log\left(exp(-y^{\left(i\right)}\left(x^{\left(i\right)}w^{T}+b\right))+1\right),$$

where the first term refers to the Ridge regularization. In this paper, the best value for the hyperparameter C>0 is found by 5-fold CV.

### 3.4. Decision Trees

Decision Trees (DT) are non-linear and non-parametric approaches constructed from a set of conditions organized in a hierarchical structure according to an index related to entropy (e.g., the Gini index) [46]. In this work we have considered the Classification and Regression Trees (CART), widely used in the literature [47]. In CART, the feature providing the highest Gini index [22] is chosen to obtain a new branch in the tree, and therefore, new nodes.

When a new node is created, the associated region in the feature space is split in two disjoint regions by a linear boundary. Each of the two new regions are associated with the majority class among the training samples encompassed by that region. When new branches and nodes are created, the tree depth increases. The final depth is determined by the terminal nodes (those with no more splitting). Note that large trees with many terminal nodes and very few samples per terminal node can lead to a limited generalization capability. The final tree structure depends on the following hyperparameters: the minimum number of samples to split a node, the maximum number of samples for a terminal node and the maximum depth. Values for these hyperparameters are chosen by 5-fold CV in this work.

### 3.5. XGBoost

XGBoost or XGB (from eXtreme Gradient Boosting) is a boosting algorithm, an ensemble technique of sequential learning. Boosting-based techniques are sequential approaches creating an ensemble of *t* sequential models ${\left\{f_{k}\right\}}_{k=1}^{t}$ (CART in this work) trained one after another, to get a robust classifier [48,49]. Each time a new model is incorporated, it is trained on the residuals provided by the previous model in the sequence. Thus, the XGB prediction for the *i*th sample $x^{\left(i\right)}$ when incorporating the *t*-th model is obtained as ${\hat{y}}_{t}^{\left(i\right)}=\sum_{k=1}^{t}f_{k}\left(x^{\left(i\right)}\right)={\hat{y}}_{t-1}^{\left(i\right)}+f_{t}\left(x^{\left(i\right)}\right)$. In other words, each time a new tree is sequentially incorporated, the XGB prediction is obtained by maintaining predictions provided by previous models and adding the residual prediction provided by the new model (see [50] for details).

When training XGB, the function to be optimized is a regularized function penalizing complex models by taking into account the complexity $\Omega (f_{k})$ for each tree. It is given by:

(4) $$L\left(\phi \right)=\sum_{i=1}^{N_{t}}l\left({\hat{y}}_{t}^{\left(i\right)},y^{\left(i\right)}\right)+\sum_{k=1}^{t}\Omega \left(f_{k}\right),$$

where *l* is a derivable convex loss function (the binary cross entropy function in this work) measuring the difference between the prediction ${\hat{y}}_{t}^{\left(i\right)}$ provided after incorporating the *t*-th tree and the target output $y^{\left(i\right)}$ for the *i*th sample.

### 3.6. Artificial Neural Networks

Artificial Neural Networks (ANNs) are conventional ML approaches which have acquired great interest in recent years, mainly due to the computational advances and the potential provided in many areas [51]. In the same way as biological neurons, the ANNs have a hierarchical structure with interconnected artificial neurons, organized in layers. When only one neuron is used, the architecture is named Single-Layer Perceptron (SLP) [52]. The SLP allows us to address binary classification tasks when both classes are linearly separable [53], computing the output as $f(xw^{T}+b)$, where *f*(.) is usually a sigmoid function.

The SLP implements a simple linear classifier, i.e., just one neuron, with parameters *b* and w to be learned. When the neurons are organized in layers and interconnected by a set of weights, a new architecture called Multi-Layer Perceptron (MLP) is created. The MLP is considered as a universal approximator [13] because it allows to model any relationship between a set of input features and the output whenever the MLP architecture is complex enough and the training set is adequate to determine the MLP parameters.

The MLP learning process is performed to find suitable values for the weights associated to each neuron. Training is conducted by optimizing a cost function by gradient-based approaches [54]. In this paper, we also use the binary cross entropy cost function [55], as in XGB.

## 4. Database Description

For this study, anonymized clinical data provided by the University Hospital of Fuenlabrada (UHF) in Madrid, Spain, have been analyzed. More specifically, we consider demographic and clinical information of 2600 patients admitted to the ICU during a 13-year period, from 2004 to 2016 (both inclusive). Note that different clinical episodes may be carried out for the same patient. In total, 3013 different clinical episodes are analyzed: 2743 corresponds to non-MDR episodes, and 270 to MDR episodes (positive cases).

**Figure 3:**
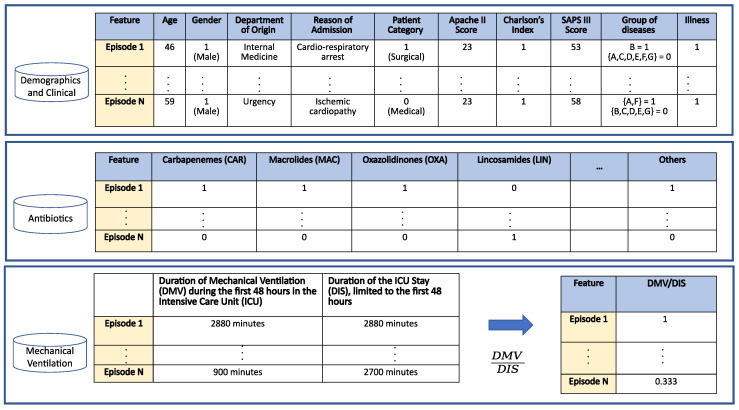
Schematic dataset description.

A total of 12 input variables are used: demographic features (*age* and *gender*), clinical features (*department of origin*, *reason of admission*, *patient category*, *Apache II score*, *Charlson’s comorbidity index*, *SAPS III score*, *group of diseases* and *illness*), the *antibiotics* given to the patient and *the percentage of time the patient was assisted with mechanical ventilation during his/her first 48 h in the ICU*. See Figure 3 for details.

**Figure 4:**
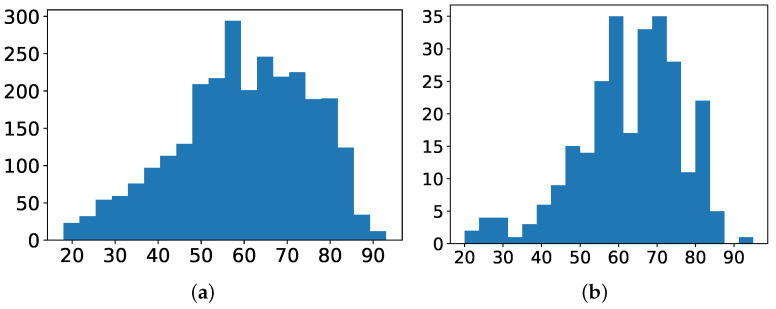
Histogram of *age* for: (**a**) non-MDR patients; and (**b**) MDR patients.

Firstly, **demographic features** are described:

- *Age*: Numerical variable referred to the age of the patient at the time of the episode. Figure 4a shows the histogram of age for patients with non-MDR episodes, whereas Figure 4b is for patients with MDR episodes. The average age for patients with MDR episodes is about 63 years, while for non-MDR ones is 60 years.
- *Gender*: Binary variable indicating whether the gender of the patient is female or male. Among the 1159/1854 episodes associated with women/men, only about 9% of the episodes associated to each gender correspond to MDR patients during the first 48 h from ICU admission.

Secondly, we describe the **clinical features** considered in this work. Since most of them are categorical, we transform them into binary features (categories) by One-Hot-Encoding [56].

**Figure 5:**
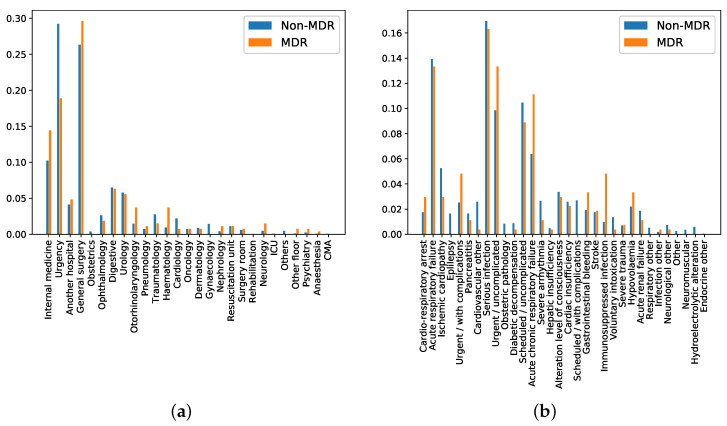
(**a**) Rate of episodes for each *department of origin*, normalized for non-MDR and MDR patient episodes; (**b**) rate of episodes for each *reason of admission*, normalized for non-MDR and MDR patient episodes.

**Figure 6:**
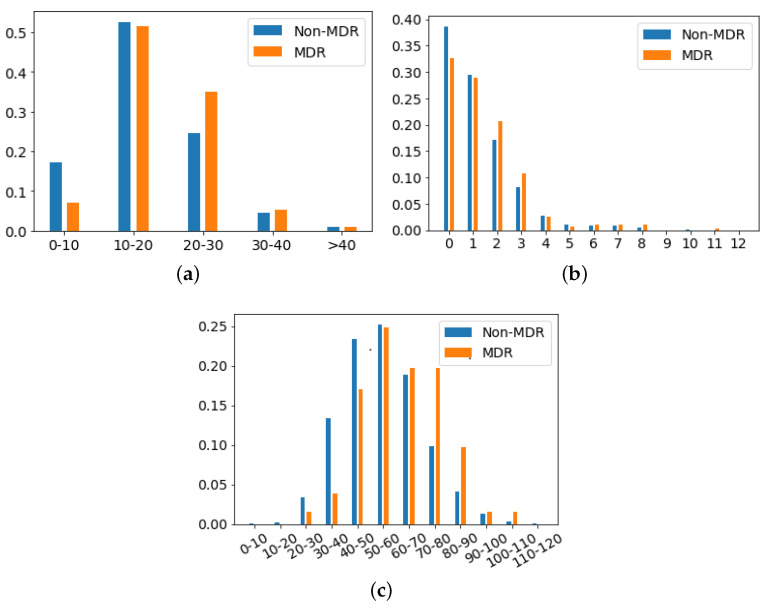
Rate of patient episodes for both MDR and non-MDR when three clinical scores are considered: (**a**) *Apache II*, (**b**) *Charlson* and (**c**) *SAPS III*.

**Figure 7:**
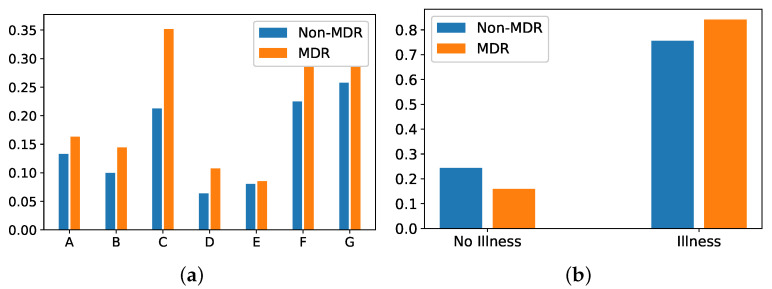
Rate of MDR and non-MDR patient episodes associated with: (**a**) each group of diseases; (**b**) illness presence.

- *Department of origin*: Categorical feature indicating the service where the patient was admitted before his/her admission to the ICU. This feature contains 27 categories (see Figure 5a), being ‘general surgery’ and ‘emergency’ the most frequent ones. It is also remarkable that the department of origin with higher rate for MDR episodes is ‘general surgery’, while it is ‘emergency’ for non-MDR episodes.
- *Reason of admission*: Categorical feature indicating the main reason for the ICU admission. It contains 32 categories, shown in Figure 5b. The categories named ‘Serious infection’ and ‘Acute respiratory failure’ are the most frequent reasons for ICU admission, both for MDR and non-MDR episodes.
- *Patient Category*: Binary feature with values associated with ‘Surgical’ and ‘Medical’, identifying whether the patient was admitted or not in the ICU just after a surgery. In our database, 40.14% of MDR episodes are ‘Surgical’, while this percentage is 44.81% for non-MDR episodes.
- *Apache II Score*: Clinical score provided by a disease severity classification system named Apache (Acute Physiology and Chronic Health Evaluation), used in the ICU [57,58]. Higher scores of Apache II are associated with a higher risk of death. In our database, the average ± standard deviation of *Apache II Score* for MDR patient episodes is 19.17 ± 6.91, while it is 17.43 ± 7.66 for the non-MDR patient episodes. This can be visually checked in Figure 6a, which shows the distribution of values per each kind of episode.
- *Charlson’s comorbidity index*: Clinical score used to predict the ten-year mortality according to the age and comorbidities of the patient. In our database, the Charlson’s average and standard deviation is 1.44 ± 1.65 for MDR patient episodes, and 1.24 ± 1.52 for the non-MDR patient episodes (see the values distribution in Figure 6b).
- *SAPS III*: A score used to estimate the probability of mortality risk based on data registered during the first 24 h of the patient admission in the ICU [59]. Higher values of SAPS III (Simplified Acute Physiology Score III) are associated with higher mortality rates. Most values of SAPS III are between the scores 10 and 20 (51.6% of total MDR patient episodes and 52.6% of total non-MDR patient episodes). It may be remarkable that the percentage of MDR episodes is higher than that of non-MDR ones (35.0% versus 25.7%) when the SAPS III score increases. For low SAPS III scores, ratios are reversed: 0.1% for MDR versus 17.3% for non-MDR.
- *Group of diseases*: Categorical feature indicating the type of clinical comobordities a patient can suffer from. In this work, seven groups related with different diseases were considered: group A (related to cardiovascular events); group B (kidney failure, arthritis); group C (respiratory problems); group D (pancreatitis, endocrine); group E (epilepsy, dementia); group F (diabetes, arteriosclerosis); and group G (neoplasms). Figure 7a shows the corresponding rate distribution for MDR and non-MDR patient episodes.
- *Illness*: Binary feature indicating whether the patient presents at least one disease according to the variable *Group of diseases*. We show in Figure 7b the distribution of this variable for MDR and non-MDR patient episodes. Note that the illness rate is higher for patients who will develop MDR.

**Figure 8:**
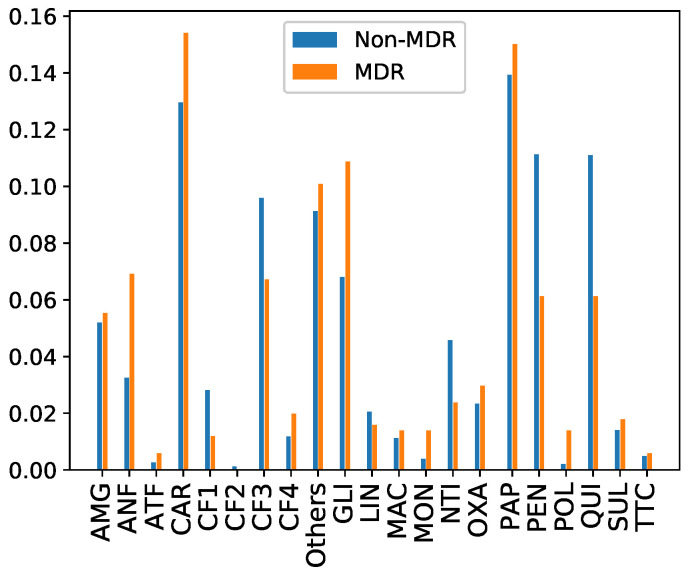
Rate of MDR and non-MDR patient episodes per family of antibiotics administered during the first 48 h.

Thirdly, **antibiotics administered in the first 48 h from the ICU admission** are considered. In this paper, antibiotics are grouped according to the family they belong to. In particular, 21 families can be distinguished: Aminoglycosides (AMG), Amphenicols (ANF), Antifungals (ATF), Carbapenemes (CAR), Cephalosporins 1st generation (CF1), Cephalosporins 2nd generation (CF2), Cephalosporins 3rd generation (CF3), Cephalosporins 4th generation (CF4), Glycopeptides (GLI), Lincosamides (LIN), Macrolides (MAC), Monobactamas (MON), Nitroimizadols (NTI), Oxazolidinones (OXA), Broad-spectrum penicillins (PAP), Penicillins (PEN), Polymyxins (POL), Quinolones (QUI), Sulfamides (SUL), Tetracyclines (TTC) and those not considered in any of the previous families (Others). Figure 8 shows that the most common families in our database are PAP and CAR, with a higher rate among MDR episodes. However, CF3, PEN and QUI present a higher rate for non-MDR episodes.

The last variable considered in this work is the ratio among the interval of time the patient has been with **mechanical ventilation** and the time interval the patient has been in the ICU, with both intervals limited to the first 48 h in the ICU. Our analysis provides that, on average, patients with MDR episodes were assisted with mechanical ventilation during 44% of their ICU stay length (limited to the first 48 h). Patients with non-MDR episodes were less assisted with mechanical ventilation, approximately during the 39% of their length of stay (again, limited to the first 48 h).

## 5. Experiments and Results

We present first the risk factors identified according to the FS methods described in Section 2. After that, we summarize and discuss the performance of several ML models when considering the selected features.

### 5.1. Identification of Relevant Risk Factors

#### 5.1.1. Based on Proportion and Median Tests

**Figure 9:**
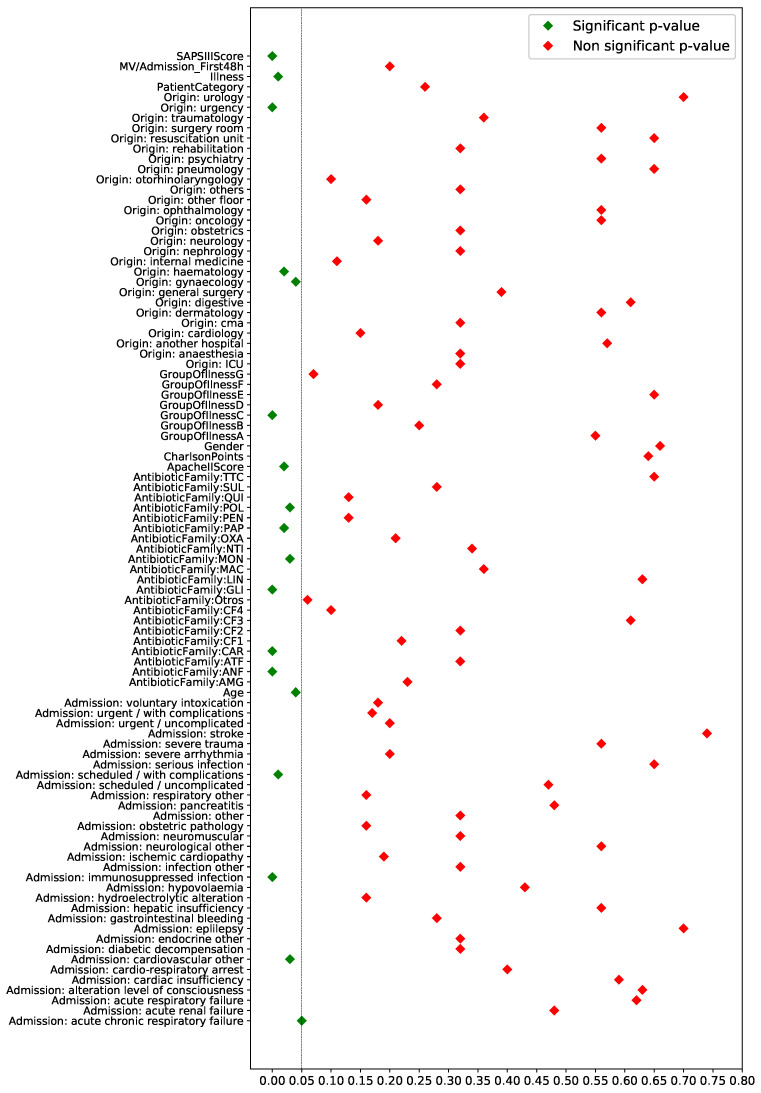
Average of the *p*-values for the 95 initial features when considering bootstrap and the proportion/median test for binary and numerical features, respectively, with a significance level of α = 0.05.

We start by presenting the risk factors obtained when considering bootstrap (*R* = 3000 resamples) for computing the average of the *p*-values when the proportions and median tests are applied. We determine a significance level of α = 0.05. Figure 9 shows in green (red) the average of the *p*-values for the selected (non-selected) features. Note that only 18 risk factors were selected as statistically significant, among them: *SAPS III Score*, the *age* of the patient and the *reason of admission referred to acute chronic respiratory failure*. In other words, 81% of the original risk factors were discarded following this procedure.

#### 5.1.2. Based on Mutual Information

**Figure 10:**
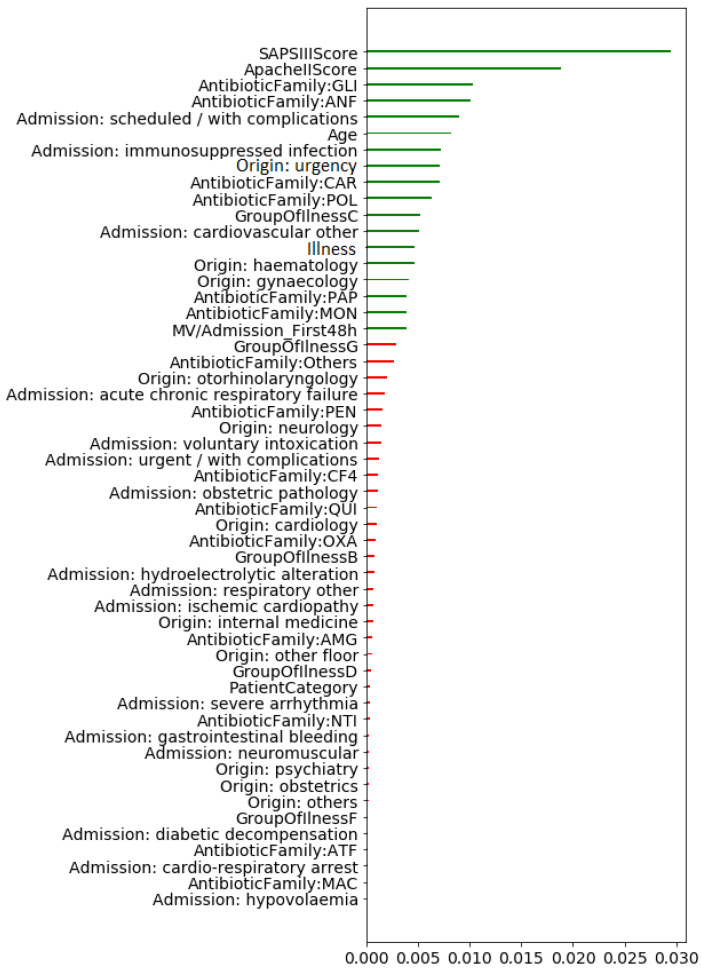
Averaged mutual information (MI) values when bootstrapping the patient episodes for each feature. Features with very low MI values are not shown here. In green, the 18 features with higher MI values.

We leverage the MI criterion to find risk factors for MDR patient episodes when considering bootstrap. The averaged MI values are shown in Figure 10, representing in green the 18 features with higher average values. In general, selected risk factors are consistent with those obtained with the proportions/median test. There is only one mismatch: the *acute chronic respiratory failure feature*, which was previously selected and it is now replaced by *MV/admission: First 48 h*.

#### 5.1.3. Based on Confidence Intervals

**Figure 11:**
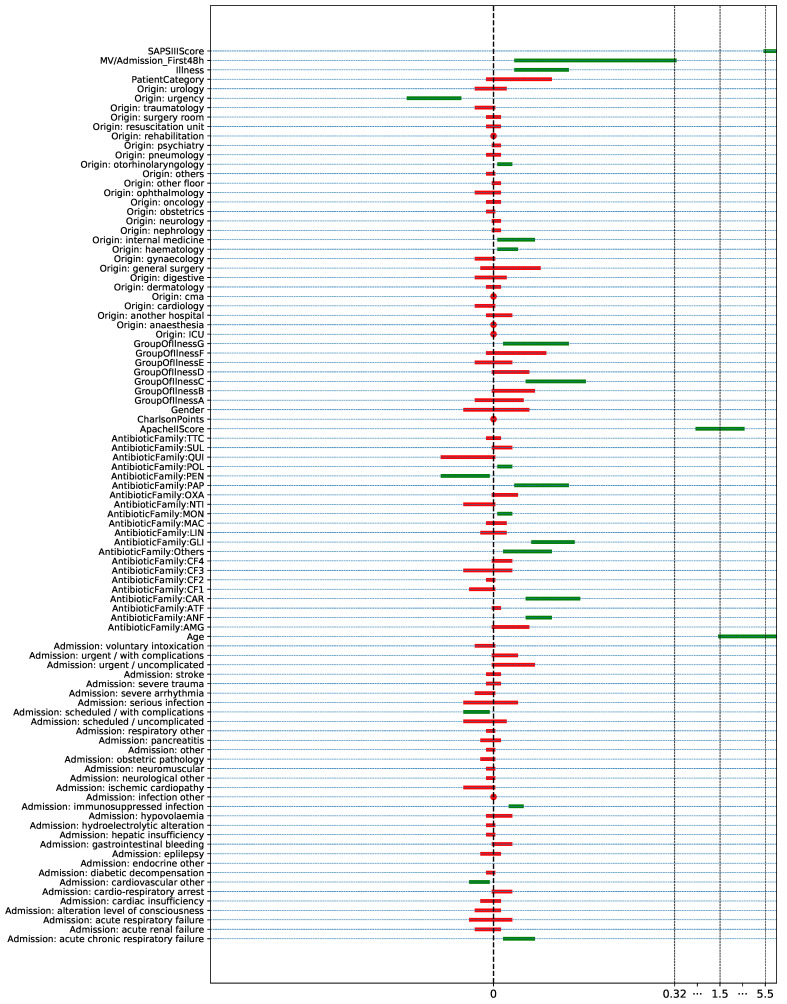
CI for numerical features ($CI_{\Delta m}$ ) and for binary features ($CI_{\Delta p}$) when bootstrapping MDR and non-MDR patient episodes. The selected features are represented in green.

The last procedure considered in this work to identify risks factors is based on the CI of the difference between the proportions/median of each feature for MDR and non-MDR episodes (see Section 2.4). Figure 11 shows the 95% of the CI when bootstraping the episodes associated with each feature. We represent in green the 23 features such that the corresponding CI does not overlap the zero value. Though the results are consistent with those obtained using both the *p*-value (proportions/median test) and the MI, certain differences can be found. The feature *Origin: gynecological*, selected with the proportions test and MI, is not selected when using the CI criterion, since it overlaps the zero value. However, the variables *MV/Admission: First 48 h*, *Origin: otorhinolaryngological*, *Origin: internal medicine*, *GroupOfIllnessG*, *AntibioticFamiliy: PEN* and *AntibioticFamiliy: Others* are statistically significant using the CI criterion.

**Figure 12:**
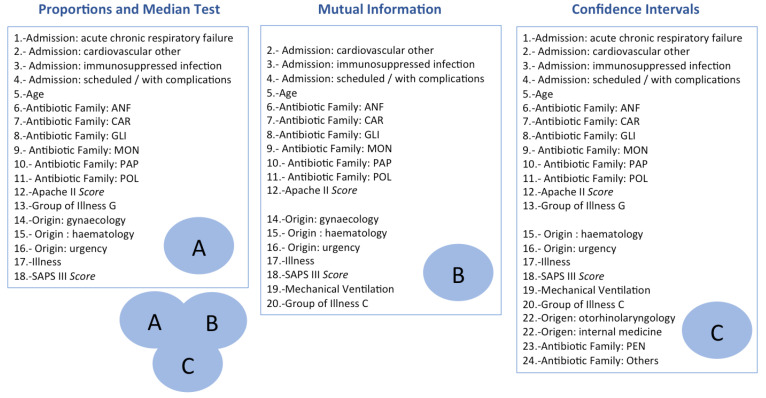
Description of the selected features with the three Feature Selection (FS) methods: Proportions and Median Test, MI and CI. The final set of selected features is the union of the features identified with each FS strategy.

According to the previous strategies, we identify the union of all the risk factors identified by the proportions/median test, MI and CI as the final subset of selected features, providing 24 features in total (see Figure 12 for details). Since the use of highly correlated features can worsen the model performance [60], we compute the Pearson [61] and the Spearman correlation coefficient [62] for numerical and binary features, respectively. In absolute values, the highest correlation coefficients were 0.59 between the *Apache II Score* and the *SAPS III Score* features, and 0.60 between *Origin: general surgery* and *Patient Category*. According to these correlation values, we maintain the 24 previously selected features.

### 5.2. Artificial Intelligence Models to Predict MDR in the ICU

We consider five ML schemes, namely LR, DT, XGB, SLP and MLP. For each scheme, two strategies to deal with imbalanced classes are explored: undersampling and weighted cost (including a priori information in the cost function). These schemes are trained and evaluated following the considerations described in Section 3.1. The hyperparameters configuration has been selected as the one providing the highest performance in terms of AUC-Score by following a 5-fold CV approach. Results in terms of accuracy, sensitivity, specificity and AUC-Score are presented in Table 1.

In general terms, we can conclude that linear models (LR and SLP) provide the best results for accuracy, specificity and AUC. Regarding sensitivity, DT achieve on average better performance, though the standard deviation is higher than that of linear models. As for the class-balancing strategy, a better performance is provided by models when considering a higher number of training samples (no undersampling). This result stresses the difficulty for creating models when learning from a limited number of samples in relation to the number of features.

## 6. Discussion

There are several studies supporting that MDR continues growing worldwide [63,64]. It poses a major obstacle in the treatment of infectious diseases, considerably increasing the length of the hospital stay, the mortality rate and the involved costs. Owing to the health conditions of the ICU patients, the use of antibiotics is quite frequent and the duration of the treatments is long [65]. It is therefore essential to anticipate the development of MDR with the aim of isolating the ICU patient as soon as possible, thus avoiding crossed transmission to other patients in the ICU.

Since it is still challenging to anticipate results provided by the microbiology laboratory during the first 48 h from the ICU admission, we propose in this paper a strategy based on FS and ML techniques. Specifically, our goal is to identify risk factors and create a predictive data-driven model to get insights of MDR and to support clinical decision-making. The early identification of patients with high risk to develop MDR may provide useful knowledge for all stakeholders in the health care process. As an immediate advantage, it could help to determine the appropriate antimicrobial therapy, thus decreasing the death rate, the workload and the number of infections in the ICU.

The risk factors identified in this paper with filter methods are in accordance with the clinical knowledge. In particular, the *SAPS III* and *Apache II* scores, as well as the *patient age* and the *department of origin before ICU admission* are helpful to discriminate between MDR and non-MDR patient episodes. For future work, we propose to explore the potential of wrapping and ensemble methods such as Recursive Feature Elimination (RFE) or Least Absolute Shrinkage and Selection Operator (LASSO) [11,41]. The use of these approaches, which combine the FS process with the learning procedure, may enhance the model performance as well as the identification of relevant features. In this line, a recent study [60] has proposed a statistical approach to provide a better characterization of redundant features and to distinguish informative features from the noisy ones.

Furthermore, the performance obtained when applying ML models may be improved by increasing the number of patients, their diversity and other features which may be relevant for this setting. Among these features, the patient’s bed may be of interest because MDR could be transmitted to the closest patients, or even variables at ICU level as the total number of nurses working in the unit. Future work also concerns deeper analysis of other artificial intelligence approaches such as Support Vector Machines (SVM) [41] or Gaussian Processes [66]. ML could be useful to predict MDR both using different data source individually (e.g., antibiotics or vital signs) or leveraging the potential of combining them [10]. Further research in this line may be considered to evaluate the suitability of the applied ML according to the nature of the features used for learning.

## Figures and Tables

**Table 1:** Mean ± standard deviation of the performance (accuracy, sensitivity, specificity, AUC) on 50 test sets when training different ML models using two class-balancing strategies. The highest average performance for each figure of merit is in bold.

Model	Class-Balancing Strategy	Accuracy	Sensitivity	Specificity	AUC
**LR**	*Undersampling*	0.618 ± 0.046	0.595 ± 0.077	0.646 ± 0.071	0.620 ± 0.047
	*Weighted cost*	**0.661 ± 0.015**	0.614 ± 0.069	**0.665 ± 0.019**	**0.640 ± 0.031**
**DT**	*Undersampling*	0.568 ± 0.049	0.559 ± 0.128	0.581 ± 0.134	0.570 ± 0.048
	*Weighted cost*	0.558 ± 0.100	**0.628 ± 0.132**	0.551 ± 0.122	0.590 ± 0.027
**XGB**	*Undersampling*	0.587 ± 0.047	0.574 ± 0.077	0.607 ± 0.077	0.590 ± 0.047
	*Weighted cost*	0.575 ± 0.221	0.602 ± 0.204	0.572 ± 0.261	0.587 ± 0.048
**SLP**	*Undersampling*	0.621 ± 0.045	0.599 ± 0.070	0.649 ± 0.069	0.624 ± 0.045
	*Weighted cost*	0.660 ± 0.015	0.616 ± 0.067	0.664 ± 0.018	**0.640 ± 0.031**
**MLP**	*Undersampling*	0.581 ± 0.050	0.575 ± 0.100	0.595 ± 0.099	0.585 ± 0.049
	*Weighted cost*	0.639 ± 0.039	0.614 ± 0.086	0.642 ± 0.046	0.628 ± 0.036
